# Decomposing biodiversity data using the Latent Dirichlet Allocation model, a probabilistic multivariate statistical method

**DOI:** 10.1111/ele.12380

**Published:** 2014-10-17

**Authors:** Denis Valle, Benjamin Baiser, Christopher W Woodall, Robin Chazdon

**Affiliations:** 1 School of Forest Resources and Conservation, University of Florida136 Newins-Ziegler Hall, Gainesville, FL, 32611, USA; 2 Wildlife Ecology and Conservation, University of Florida110 Newins-Ziegler Hall, Gainesville, FL, 32611, USA; 3 United States Forest Service, Northern Research Station1992 Folwell Avenue, St. Paul, MN, 55108, USA; 4 Department of Ecology and Evolutionary Biology, University of Connecticut75 N. Eagleville Road Unit 3043, Storrs, CT, 06269-3043, USA

**Keywords:** Biodiversity data, cluster analysis, community ecology, Latent Dirichlet Allocation, multivariate statistics, text-mining

## Abstract

We propose a novel multivariate method to analyse biodiversity data based on the Latent Dirichlet Allocation (LDA) model. LDA, a probabilistic model, reduces assemblages to sets of distinct component communities. It produces easily interpretable results, can represent abrupt and gradual changes in composition, accommodates missing data and allows for coherent estimates of uncertainty. We illustrate our method using tree data for the eastern United States and from a tropical successional chronosequence. The model is able to detect pervasive declines in the oak community in Minnesota and Indiana, potentially due to fire suppression, increased growing season precipitation and herbivory. The chronosequence analysis is able to delineate clear successional trends in species composition, while also revealing that site-specific factors significantly impact these successional trajectories. The proposed method provides a means to decompose and track the dynamics of species assemblages along temporal and spatial gradients, including effects of global change and forest disturbances.

## Introduction

Multivariate analyses in community ecology were initially applied to vegetation data (Williams & Lambert 1959; Whittaker 1967) and since have been applied to a broad range of ecological communities from benthic invertebrates (Clarke 1993) to microbes (Ramette 2007). While the multivariate toolbox that community ecologists have utilised includes a breadth of analyses (Gauch 1982; McCune *et al.* 2002; Borcard *et al.* 2011; Legendre & Legendre 2012), these tools do not always conform to the conceptual models that ecologists use. For instance, a common conceptual model for variation in species abundance posits that a particular set of species might be gradually replaced by another set of species along an environmental gradient, resulting in an intermediate transition area with a mixed composition (i.e. not dominated by any given community). Unfortunately, the multivariate clustering methods commonly employed to identify these sets of species (e.g. hierarchical or k-means cluster analysis) are best suited for abrupt changes in species compositions and fail to adequately represent the gradual transitions described above.

For the first time, we propose the use of a probabilistic model called Latent Dirichlet Allocation (LDA) for biodiversity data. More specifically, when we refer to biodiversity data, we mean a matrix with the abundance of each species at each site. This method generates biologically interpretable results because it decomposes each sampling unit into distinct component communities; and characterises each of these component communities in terms of the relative abundance of species. Furthermore, the model adequately represents the uncertainty associated with its estimates and properly handles missing data. LDA was originally proposed in 2003 for applications involving text-mining (Blei *et al.* 2003) but over time this model has become a key tool for the machine learning community, being applied to a wide range of problems such as fraud detection (Xing & Girolami 2007), digital image analysis (Vaduva *et al.* 2013) and bioinformatics (Liu *et al.* 2010).

We start by providing a succinct description of LDA. Then, we illustrate its use with simulated data and contrast the inference provided by our method with that from standard clustering tools used for the analysis of biodiversity data. Finally, we apply LDA to two real-world applications: spatial and temporal patterns in temperate forest species composition and inferred temporal patterns in secondary Neotropical forest succession. These applications illustrate the fresh insights that can be gained through the use of LDA. We conclude with a discussion of the limitations of this method and suggestions of topics for future research.

## Material and methods

### Analogy between text-mining and analysis of biodiversity data

The LDA model was originally devised for text-mining and it is still widely used in this area. One of the goals in text-mining is to determine the underlying topics (e.g. ‘genetics’, ‘neurophysiology’, ‘laser’, etc.) of documents in a corpus based on the frequency of words used in each document. Each topic is characterised by a distribution over words. For instance, the topic ‘genetics’ might have a high frequency of words like ‘DNA’, ‘chromosomes’ and ‘mutation’, whereas other words are likely to have much lower frequency in this topic, such as ‘foliage’ and ‘landscape’.

The relationship between text-mining and the analysis of biodiversity data is straightforward, although previously unacknowledged. We want to characterise each sampling unit (document) in terms of its component communities (topics). Each component community (topic) corresponds to a distribution over species (words). For example, an early successional forest community might have a high relative abundance of fast-growing species that are intolerant to shade, whereas other species would have a lower relative abundance. The data that we require for this model consist of a matrix of sampling unit by individual species abundance (document by word table). To our knowledge, the analysis of biodiversity data with this text-mining tool (LDA) is a novel application.

### Model characterisation

**Figure 1:**
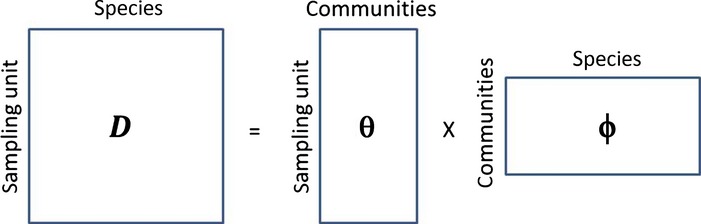
Conceptual matrix factorisation implied by the Latent Dirichlet Allocation model (adapted from Steyvers & Griffiths 2007). The matrix containing the relative abundance data D is factorised into a matrix *θ* that describes the relative abundance of each component community in each sampling unit and a matrix *ϕ* that describes the relative abundance of each species in each component community.

In this model, each sampling unit (e.g. a site, a river, a field plot, an organ, etc.) contains information regarding the taxonomic identity (e.g. species or operational taxonomic unit) of individuals within this unit. Each sampling unit *l* (*l *=* *1,…,*P*) has an associated vector of probabilities ***θ***_*l*_ = [*θ*_1*l*_, …, *θ*_*cl*_], where 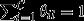, which describes the relative abundance of component communities 1,…,*C* at this site (i.e. the relative frequency of individuals from each of these communities). Similarly, each component community *j* is characterised by a vector of probabilities ***ϕ***_*j*_ = [*ϕ*_1*j*_, …, *ϕ*_*sj*_], where 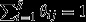, which describes the relative abundance of species 1,…,S in this component community. These parameters define the conceptual factorisation depicted in Fig. 1. In this figure, the matrix containing the relative abundance data **D** is factorised into a matrix ***θ*** that describes the relative abundance of each component community in each sampling unit and a matrix ***ϕ*** that describes the relative abundance of each species in each component community.

**Figure 2:**
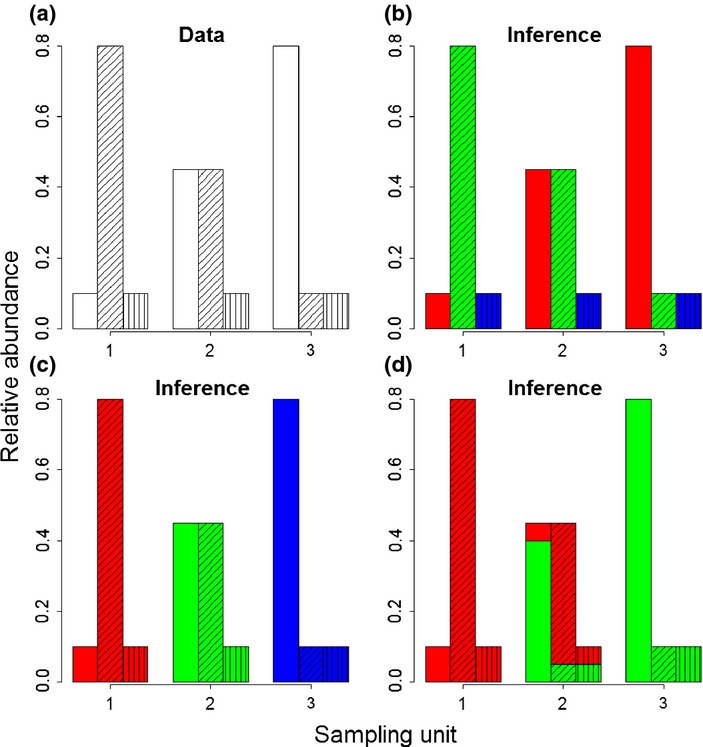
Example of the inference provided by the Latent Dirichlet Allocation model. We provide a simple example involving three species (no lines, diagonal lines and vertical lines) and three sampling units (three groups of vertical bars). Data are shown in panel (a) and the resulting inference from the LDA model in the remaining panels. Panels (b and c) assume three component communities (colour coded as red, green and blue) while panel (d) assumes only two component communities (colour coded as red and green).

A simple example helps to illustrate the type of outcomes this model can provide. Say we have three species (bars with no lines, diagonal lines and vertical lines in Fig. 2a) and three sampling units (three groups of vertical bars in Fig. 2a). In this example, the data matrix in terms of relative abundance is 
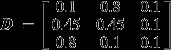
. If we assume three component communities (C = 3), the algorithm might perfectly fit the data in two ways:

1. Each component community (red, green and blue) is comprised of a single species, which implies that 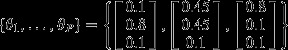 and 
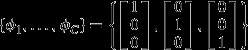
 (Fig. 2b); or
2. Each sampling unit is composed of a single component community (red, green and blue), in which case
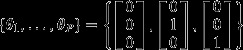
 and 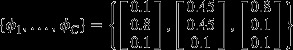 (Fig. 2c).

Either way, these results would not be very enlightening. However, if we assume two component communities (C = 2), then the algorithm is still able to perfectly fit the data by assuming that sampling unit one is composed solely by the red component community, sampling unit three is composed only by the green component community and sampling unit two is a 50–50 mixture of the red and green component communities (Fig. 2d). This would imply that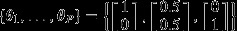 and 
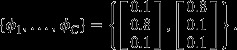


This last example illustrates that sampling units can be mixed (i.e. have individuals coming from different component communities). The ability to represent a sampling unit as being composed by multiple component communities (e.g. Fig. 2d) is a distinct feature of our method since standard multivariate techniques typically assign each sampling unit to a single group. This feature is important because it allows for the model to represent both sharp and gradual changes in species composition (e.g. due to environmental or temporal change). Additional details regarding LDA (e.g. the data-generating model, how it was fit and the folding-in and data imputation operations) are available in the online supporting information.

An important decision refers to the number of component communities. Similar to most clustering methods (Jain 2009), the number of component communities has to be chosen *a priori*. Our approach is to fit models with different number of component communities and choose the best model based on a model selection criterion such as the Akaike information criterion (AIC), which balances model fit with model complexity. To calculate AIC, we used the maximum *a posteriori* probability estimates which, given the uniform (within the simplex) priors adopted in our analysis, approximate the maximum likelihood estimates.

The development and application of clustering methodologies have a long history, with thousands of clustering algorithms developed and applied across multiple scientific disciplines (Jain 2009; Fortunato 2010). As a result, we simply focus on clarifying what sets LDA apart from existing clustering methods. The originality of LDA is that it combines several features into a single tool. For instance, LDA allows for sampling units to be composed of multiple component communities (somewhat similar to fuzzy clustering), it accounts for missing data and provides uncertainty estimates (similar to probabilistic clustering). A more detailed comparison of LDA with existing clustering methods is given in the online supporting information.

### Simulations

**Figure 3:**
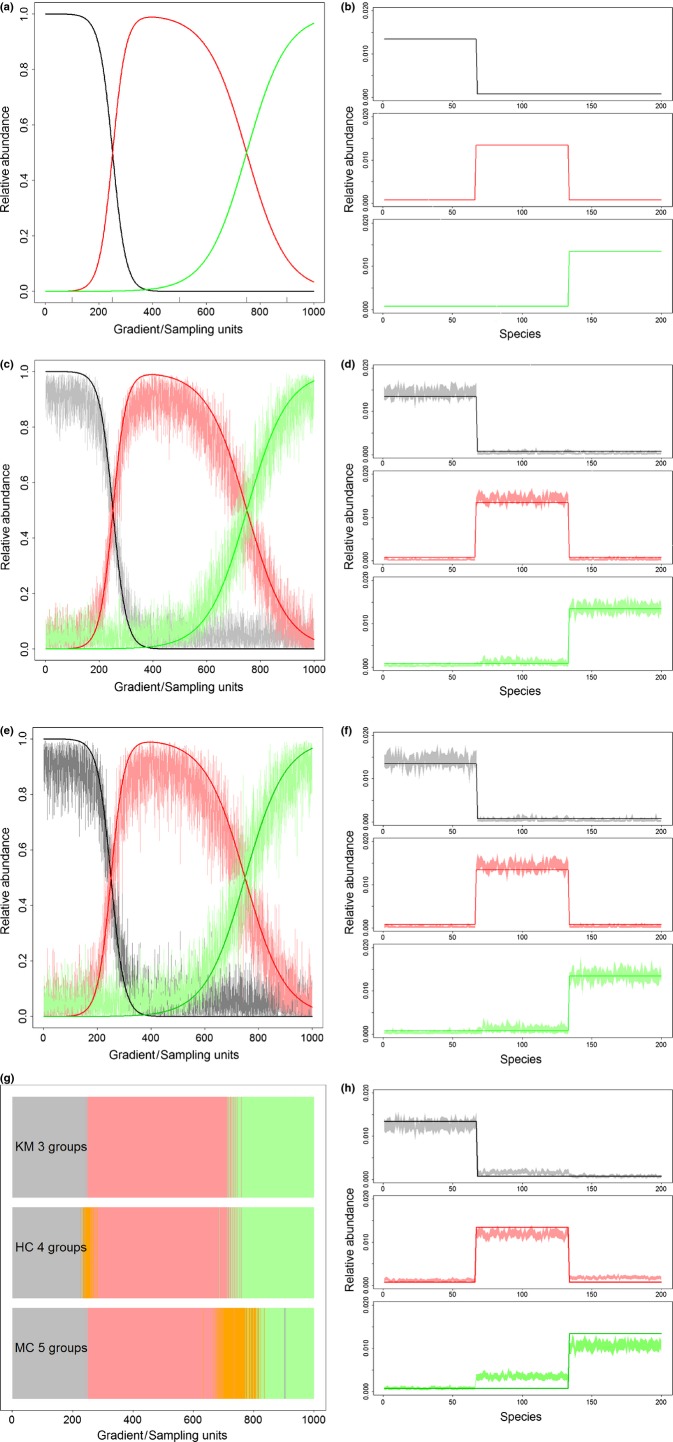
Simulated data. We assume three component communities (black, red and green) and display in Panel (a) how their relative abundances (*θ*_*l*_, *y*-axis) changes along a gradient (*x*-axis). Panel (b) shows the relative abundance (*y*-axis) of each of the 200 species (*x*-axis) for these three component communities (*ϕ*_*j*_). Panels (c and d) show inference from LDA based on the complete data set, where 95% credible intervals for the estimated parameters are depicted in lighter colours. Panels (e and f) show LDA results for the data set with missing observations. Panel (g) shows inference from k-mean clustering (KM), hierarchical clustering (HC) and model-based clustering (MC) on the complete data set. The designated cluster of each sampling unit is shown with a vertical colour-coded line. Panel (h) shows the relative abundance of species in each cluster based on the KM results with three groups.

We illustrate model performance with an example based on simulated data, which although simplistic, clearly shows the differences between the method we propose and three more traditional multivariate methods. We simulate data showing gradual changes in the proportion of three component communities (colour coded as black, red and green; *y*-axis in Fig. 3a) along a single axis, which could represent time, latitude or any environmental gradient (*x*-axis in Fig. 3a). We place 1000 sampling units systematically distributed along this gradient. Thus, Fig. 3a displays the vectors ***θ***_*l*_ for *l *=* *1,…,1000 and reveals that the black component community gradually gives way to the red component community which eventually is replaced by the green component community as we move along the gradient from sampling unit 1 to 1000. We assume 200 species and 100 individuals per sampling unit. Despite these three component communities sharing all species, we chose to have very different relative abundances of species in each component community. Fig. 3b displays the vectors ***ϕ***_*j*_ for j = 1,2,3 showing that species 1–67, 68–133 and 134–200 are more abundant in communities 1, 2 and 3 respectively. We fitted multiple LDA models by varying the number of component communities from 2 to 10 and chose the best model based on AIC.

Missing data are common when combining multiple biodiversity surveys or when combining biodiversity with environmental indicator species surveys (De Caceres *et al.* 2012) due to differences regarding the list of taxa that are monitored. Thus, we also explored the performance of LDA in the presence of missing data. To this end, we used the same parameters and data as described previously but we assumed that 50% of the sampling units had missing data for 50% of the species. Sampling units and species with missing data were selected at random.

We compared results from LDA on the complete simulated data with that of four commonly used clustering tools, namely: (1) hierarchical clustering (HC) with analysis of similarity permutation tests (ANOSIM; Clarke 1993), (2) HC with multi-response permutation procedure (MRPP; reviewed by McCune *et al.* 2002), (3) k-means (KM) clustering and (4) model-based clustering (MC). Both LDA and MC were run 10 times to assess the robustness of their results.

### Case study 1: Spatial distribution of tree communities in the Eastern United States

The first case study uses LDA to explore spatial and temporal patterns in tree communities across the eastern United States using tree data [diameter at breast height (DBH) > 12.7 cm] from the Forest Inventory Analysis (FIA) program (Woudenberg *et al.* 2010). These data come from 672 m^2^ plots sampled between 2008 and 2012. We selected all plots that were fully forested in the Eastern United States and that had at least 10 trees. Our final data set contained 34 174 plots (out of 86 102 plots), 219 species and 989 047 trees. To detect temporal changes, we also relied on FIA data from earlier inventories conducted in Minnesota and Indiana. These states were chosen because they had the earliest forest inventories conducted with the same measurement protocol of current FIA inventories. These earlier inventories for Indiana and Minnesota were conducted in 1998 and 1999–2003, encompassing 675 and 2013 plots respectively. We fitted multiple models to our FIA data, varying the number of component communities from 3 to 26. We then selected the optimal number of component communities based on AIC and report the results from the best model.

### Case Study 2: Secondary forest chronosequence in Costa Rica

The second case study explores secondary successional patterns of tree composition in wet tropical forests. Data for this case study come from six secondary forest and two old-growth forest sites in Costa Rica. Within each site, data were collected using 1-ha plots and are segregated by size class: sapling (1–4.9 cm DBH), small tree (5–10 cm DBH) and large tree (> 10 cm DBH). While data on small and large trees contained information on all species, the sapling data only contained information on canopy tree species. A summary of these data by plot and size class is given in Table S2.

Because we are primarily interested in the species composition of trees DBH > 10 cm, we initially perform our analysis on data from this size class. Then, using the same component communities determined by this initial analysis, we examine how the composition of the smaller sizes classes is changing, under the implicit assumption that species composition of small trees and saplings represent the future species composition of large trees. To handle the missing species in the sapling data, we used the folding-in operation in conjunction with data imputation (see the online supporting information). We fitted our model to the large tree data assuming two to seven component communities and chose the optimal number of communities based on AIC.

## Results

### Simulations

We were able to correctly identify the model with three communities as the best model in all model runs. The 95% credible interval provided by LDA suggests that this method performs well in identifying the relative abundance of the component communities in each sampling unit (Fig. 3c) and the relative abundance of species in each component community (Fig. 3d). Even in the presence of substantial amounts of missing data (50% of the sampling units had 50% of the species with missing data), LDA was still able to retrieve the true parameters, albeit exhibiting much more uncertainty than when using the complete data (Fig. 3e and f).

Overall, our results reveal that the commonly used clustering methods tend to find 3 to 5 significant clusters, often assigning sampling units that have approximately an equal share of two component communities (i.e. mixed sampling units) to distinct groups (Fig. 3g). Additional simulations confirm that these clustering methods tend to require many more groups than LDA to result in a similar fit to the data (see online supporting information), suggesting that LDA's capability to accommodate mixed sampling units results in much more parsimonious groupings, which is critical for the visualisation/interpretation of biodiversity data. The results of the k-means clustering (KM) method also reveal problems in characterising the relative abundance of species in these communities (Fig. 3h). By assigning several mixed sampling units with a large fraction of the red community to the green community (top row in Fig. 3g), KM tends to characterise the green community with a much higher relative abundance of species 68–133 (which are characteristic of the red community) than warranted (bottom row in Fig. 3h).

### Case study 1: Spatial distribution of tree communities in the Eastern United States

**Figure 4:**
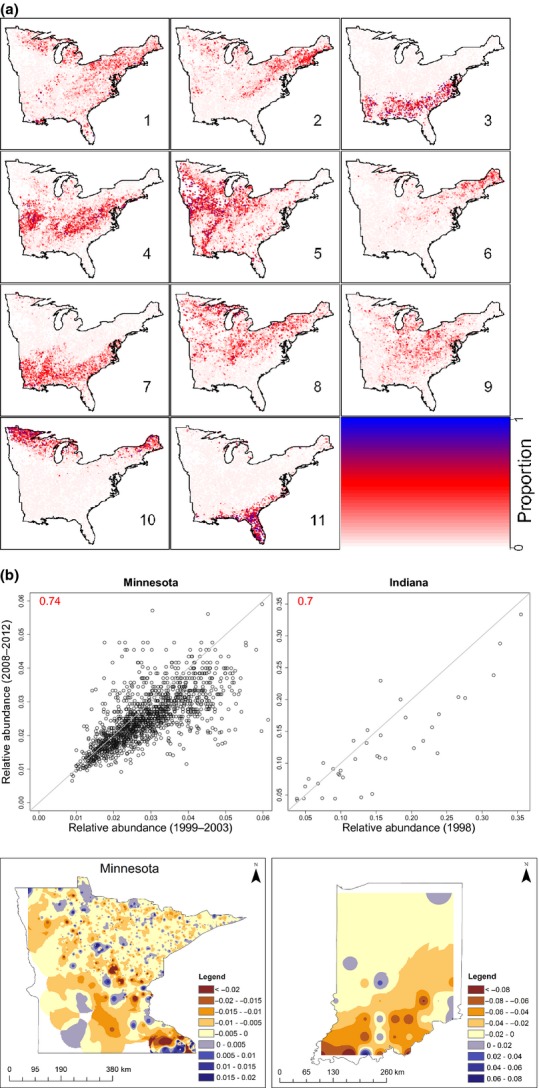
Spatial and temporal patterns for tree plots in Eastern United States. Panel (a) depicts the spatial distribution of the proportion of each component community. Each subpanel corresponds to a component community (numbers in the lower right corner, see Table S1) except for the colour key in the lower right. Panel (b) shows temporal patterns of relative abundance of the oak community in Minnesota (left subpanels) and Indiana (right subpanels). Upper subpanels show the relative abundance of community 4 in earlier and current forest inventories (numbers in red are the proportion of plots indicating a decline in relative abundance). Lower subpanels show the spatial distribution of this decline, based on an inverse-distance weighted interpolation. Data from Minnesota refer only to re-measured plots while data from Indiana were grouped into latitude–longitude bins because no plots were re-measured. Only bins with at least four plots in 1998 and 2008–2012 are used.

Our algorithm identified 11 tree component communities (the dominant characteristic species of each component communities are listed in Table S1). A map of our results indicates that several of these component communities are spatially segregated in rough latitudinal bands, as expected. Component communities that conform to this pattern were (from south to north) communities 11, 3, 7, 4, 2, 6 and 10 (Fig. 4a). On the other hand, component communities 1, 5, 8 and 9 were more dispersed spatially and did not exhibit clear latitudinal patterns. While some component communities matched well with the forest types groups adopted by FIA (e.g. component communities 3, 8 and 11), several of the other component communities did not. These results indicate that LDA can provide an alternative perspective on forest types, which may aid on the task of defining and delineating forest types. Our results also reveal how certain component communities are present throughout a much larger region, albeit not being the dominant community (e.g. component community 9 in Fig. 4a). Because forest types play a central role for the practice of forestry (e.g. for planning forest stand treatments or assessing forest resources across large-scales), it is critical that the actual relative abundance and spatial range of these forest types be correctly determined.

Determining where these forest types are changing is particularly important in the face of global change. For instance, we find a ubiquitous decline in Indiana and Minnesota of component community 4, which is dominated by oaks (*Quercus alba*,*Quercus prinus* and *Quercus velutina*) (Fig. 4b). The large-scale compositional changes documented here might be a direct consequence of fire suppression in the region, potentially being a signature of the wide-spread ‘mesophication’ process of Eastern US forests (Nowacki & Abrams 2008). These changes may also be attributed to increases in growing season moisture, which has resulted in the decline of drought-tolerant tree species such as oaks, as well as increased herbivory (McEwan *et al.* 2011). Regardless of causal factor, the ability to detect these changes using a tool that does not focus on individual species is particularly striking for Minnesota, given that component community 4 already had a very low relative abundance (< 6%) in the original 1999–2003 inventory. Because traditional clustering algorithms do not account for mixed plots, subtle changes as these are unlikely to be detectable through these more standard methods.

### Case Study 2: Secondary forest chronosequence in Costa Rica

Based on AIC, we found the optimal number of component communities to be three. Component community one is dominated by animal-dispersed and mostly large-seeded species, including three canopy palms. On the other hand, component communities two and three are dominated by a mixture of wind- and bird-dispersed species, mostly small seeded (Table S3).

**Figure 5:**
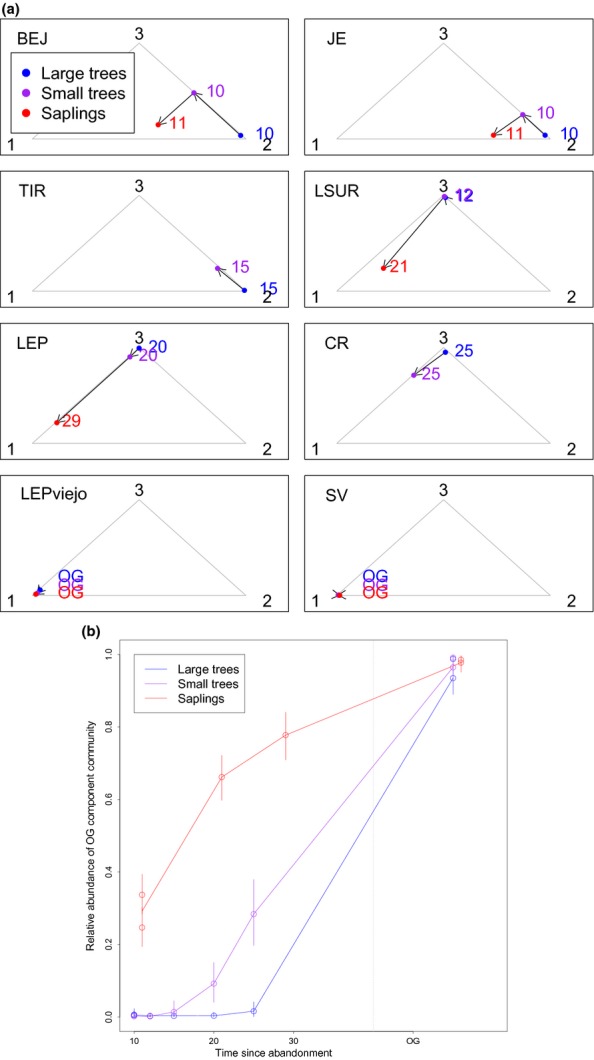
Temporal patterns inferred from the Costa Rican chronosequence based on LDA output. Panel (a) shows modelling results for each site (name of site is given on the upper left corner of each subpanel), where the relative abundance of communities was converted to barycentric coordinates to allow their display within the simplex (grey triangle). In these coordinates, observations close to triangle vertices indicate plots dominated by the respective community (black numbers). Red, purple and blue circles refer to results from sapling, small tree and large tree data respectively. Corresponding colour-coded numbers refer to time since abandonment (OG stands for old growth). Arrows indicate inferred progression in species composition. Panel (b) shows a summary of the old-growth component community patterns. Vertical bars correspond to 95% credible intervals and results are displayed separately for large trees (blue), small trees (purple) and saplings (red).

The species composition of each component community (described above) seems to agree well with expected successional patterns, but also illustrates complexity in successional trajectories. For example, our analysis reveals that the old-growth forest sites (LEPviejo and SV) are dominated by component community one, regardless of size class (Fig. 5a). Furthermore, this analysis reveals a clear successional trend of species composition changing from being dominated by component community 2, to dominated by 3 and finally dominated by 1, both within each plot for increasingly smaller trees [i.e. arrows connecting results for large trees (blue), small trees (purple) and saplings (red)] and between plots as time since abandonment increases (top to bottom panels in Fig. 5a). However, Fig. 5a also highlights substantial heterogeneity in species composition among the successional plots, even after accounting for differences in time since abandonment. For instance, LSUR clearly stands out from the other plots because it tends to have a community composition much more similar to older plots (i.e. LEP and CR) compared to other plots of similar initial age (i.e. BEJ and JE). On the other hand, TIR seems to be much more similar to the younger sites than implied by its age. These findings suggest that site-to-site differences prior to abandonment and during initial successional stages (e.g. proximity to seed sources and soil fertility) might have long-term effects on these sites, potentially overwhelming the effect of time since abandonment.

We summarise the results for the old-growth component community (community 1) in Fig. 5b to emphasise two unique features of our statistical model. The first feature is that we can only display these results because our model allows for plots to have varying proportion of each component community, rather than each plot being assigned to just one component community. The second feature refers to the 95% credible intervals that our model generates. In the absence of these intervals, it would not be possible to judge if the observed trends are really meaningful or if they are overwhelmed by the uncertainty in these parameter estimates.

## Discussion

We have described a new exploratory method to analyse biodiversity data which is based on the LDA model. This model has parameters that are biologically meaningful and straightforward to interpret. Furthermore, LDA allows for sampling units (field plots in our case studies) to be decomposed into multiple component communities, which is a key characteristic when analysing changes in species composition through time (as in the secondary succession data and FIA data) or along an environmental gradient (as in the simulated data). We emphasise that LDA can represent both gradual and abrupt transitions, whereas traditional clustering methods only represent abrupt transitions. To the extent that real assemblages are truly heterogeneous mixtures of species groups, LDA seems to be a superior method relative to existing multivariate approaches.

We have shown that LDA is able to delineate forest types in Eastern United States as well as quantify their dynamics through time. This capability to assess which tree species assemblages are expanding or shrinking in dominance is essential for managing and forecasting the forest carbon land sink and other ecosystem services. For instance, our method has detected a ubiquitous decline in the oak community in Indiana and Minnesota. While in hindsight this could have been achieved by analysing data from individual species, our results highlight that LDA is capable of identifying these patterns using data from all species jointly, even in states where the oak component community is far from being the dominant community (e.g. Minnesota). There are multiple reasons for the observed oak decline. Historically, frequent disturbances in the form of fire, grazing, farming and timber harvesting, have led to the accumulation of oaks, resulting in a pulse of oak regeneration. However, with the more recent reduction in these disturbances, particularly with fire suppression, oak regeneration has substantially declined while the extant oaks have approached physiological maturity (Spetich *et al.* 2002; Johnson *et al.* 2009; Wang *et al.* 2013). More generally, this historical trend of replacement of fire-tolerant sun-loving species by more fire-sensitive, shade-tolerant, mesophytic species, has been described as the ‘mesophication’ of forests in the Eastern United States (Nowacki & Abrams 2008).

In relation to the Costa Rican data, our results clearly show that the group of species that uniquely characterises old-growth forests is beginning to establish in second-growth forests, but are present primarily as small individuals (saplings and small trees) rather than as trees above 10 cm diameter. Our results could be interpreted to suggest that component communities 2 and 3 are early and late successional communities because they tend to dominate newer and older secondary growth sites respectively. However, this interpretation is complicated because of inherent differences between sites beyond time since abandonment, including geographical distances (i.e. plots closer to each other are likely to have similar species composition), proximity to old-growth forest as an important seed source and soil fertility. For instance, LSUR is a very young site that nevertheless is completely dominated by community 3. One potential reason could be because it is adjacent to a large extent of old-growth forest at La Selva Biological Station, which might have accelerated succession at this site. On the other hand, TIR is a site that is not that young but that has very similar species composition to the younger sites JE and BEJ, which might be because it is the most isolated site and may have severe recruitment limitation, particularly for large-seed, animal-dispersed species. Our new results highlight potential local and landscape differences that appear to influence early colonisation. Importantly, prior to this analysis, it was not apparent that second-growth forests in this region had two distinct types of tree component communities because these differences were masked by the abundance of generalist tree species. These results suggest that future research should be geared towards understanding the role of distance to seed sources in shaping species composition.

LDA is based on a fully probabilistic generative model that allows for straightforward quantification of uncertainty. This is an important characteristic of our model because it allows scientists to judge if the observed changes in species composition (e.g. due to global change) are greater than the uncertainty associated with these results. Furthermore, because of this generative model, LDA deals with missing data within a single coherent modelling framework. In other words, instead of having to devise a distinct model for imputation (e.g. multivariate regression) to then analyse the completed data, LDA assumes missing data are additional parameters to be estimated and imputes values while jointly estimating all the other parameters.

We acknowledge that the method we propose also has several limitations. One important limitation is the need to specify *a priori* the number of component communities, similar to most clustering methods. Our approach for model selection based on AIC is a viable work-around but has some issues. For instance, AIC is admittedly an unusual criterion for model selection in a Bayesian framework. Yet, it has been our experience that the more commonly used Deviance Information Criterion is not numerically very stable. We believe the use of AIC as a rough indicator of the trade-off between model complexity and goodness-of-fit is reasonable, particularly given the exploratory nature of LDA. Furthermore, model selection in a Bayesian framework is an area of active research and all information criteria have their limitations (Gelman *et al.* 2014). A different but more complicated approach is to estimate the number of communities as part of the fitting of the model, as in Teh *et al*. (2006).

It is important to acknowledge that the communities themselves may change substantially according to the number of component communities, potentially modifying the resulting interpretation and conclusions. Of course, this problem is not unique to LDA as most clustering methods have the same problem. Our opinion is that, to some extent, this is not a problem of the method *per se* but the fact that the concept of communities is (to some extent) a human construct that, nevertheless, is useful to summarise the otherwise overwhelming information in biodiversity datasets.

A second limitation of the LDA model is that many biodiversity surveys are based on occupancy, cover or incidence (presence/absence) data and thus lack abundance data. In these cases, our method cannot be applied as it currently is. A third limitation of LDA is that it implicitly conditions on the total number of species in the original data set. Thus, information on the abundance of new species has to be discarded when making predictions for new sampling units. Finally, a fourth limitation is that, similar to many existing clustering methods (e.g. model-based and k-means clustering), the results from LDA may change from one run to another. While assessing the robustness of LDA's results is important, this can be very computationally challenging for large data sets such as the FIA data.

For this paper, we have relied on our own customised Gibbs sampler. Yet, some software packages have implemented LDA and made it widely available for users. We have made R code available that relies on the ‘topicmodels’ package to analyse the simulated data in this article (see supporting information). Unfortunately, more specialised uses of the model, such as those involving the missing data imputation, may not be readily available in these packages.

We believe that one of the next steps in further developing this model for biodiversity data is to incorporate other types of information beyond abundance. In the case of forests, data from other sources, such as satellite imagery and LiDAR, could also help to characterise forest types/communities as well as interpolate results for areas without field plots. While we have used this model primarily as an exploratory pattern finding tool, another potential extension is to model the proportion of each community as a function of covariates using a Dirichlet regression, perhaps even accommodating for spatial autocorrelation, thus moving towards a more explanatory tool for biodiversity analysis. Several methodological and scientific questions could be addressed by future research. For instance, how can we account for phylogenetic and trait information when defining component communities? How do species assemblages change over time and space due to anthropogenic stressors (e.g. climate change, fire, selective logging, nitrogen deposition or presence of invasive species)? We believe that this novel model will soon become indispensable in the toolkit of ecologists.
