# Supplementary material for: Decomposing biodiversity data using the Latent Dirichlet Allocation model, a probabilistic multivariate statistical method
Source: Ecol Lett. 2014 Oct 17;17(12):1591–601. doi: 10.1111/ele.12380 (PMC4240467; doi:10.1111/ele.12380)
Supplement: Supplementary file 1 [file ele0017-1591-SD1.doc]

**Online supporting information**

**1. Comparison of LDA and other existing clustering methods / species composition analyses**

The development and application of clustering methodologies have a long history, with thousands of clustering algorithms developed and applied across multiple scientific disciplines . As a result, we do not attempt to review these methodologies here but rather focus only on the characteristics of LDA that set it apart from the more traditional clustering methods.

Biological communities are typically represented as clusters within a graph, where vertices (nodes) are the sampling units (e.g., field plots) and weighted edges indicate the strength of association among vertices (e.g., how similar these plots are in terms of species composition). Thus, the goal of identifying these communities can be directly translated to the goal of identifying clusters within a graph and this task is accomplished by devising algorithms that try to increase the total number (or strength) of edges within each cluster while decreasing the number (or strength) of edges between clusters . For these reasons, clustering algorithms are also known as graph partitioning algorithms .

Traditionally, each vertex is typically classified into a single cluster (i.e., non-overlapping communities). However, allowing for a sampling unit to be composed of more than one community (as in LDA) can be important; for instance, plots in transition zones (e.g., ecotone) are likely to be composed of overlapping communities. Alternative clustering methods exist that estimate the degree to which a vertex belongs to a given cluster but they are not commonly used in Ecology. Gaussian mixture models are also popular and, similar to fuzzy clustering, they estimate the degree/probability to which a vertex belongs to a given cluster. However, the implicit assumption behind fuzzy clustering and Gaussian mixture models is that a vertex belongs to only one cluster but that we are not necessarily sure to which. On the other hand, LDA explicitly acknowledges the mixed composition of a vertex by assuming that the individuals that are part of this vertex belong to potentially different clusters.

Another important distinction between traditional clustering methods and LDA refers to the representation of uncertainty. While some probabilistic clustering methods exist based on (often Gaussian) mixture models , most traditional clustering algorithms do not report measures of uncertainty associated with their outcomes. Yet, the reporting of uncertainty together with point estimates is a central tenet in statistics, being particularly critical when scientists are trying to detect and quantify the impact of global change on biodiversity. The reliance on a generative model by LDA and some of these other probabilistic clustering methods is especially useful when dealing with missing data because it allows for data imputation as part of the fitting algorithm, yielding uncertainty estimates that fully account for the fact that part of the data were missing.

We conducted a brief literature review on clustering algorithms used in ecology by performing a systematic search at two of the top ecology journals (Ecology Letters and Ecology). We specifically searched Google Scholar for all articles published between 2011 and 2013 that contained the terms “cluster” *and* “analysis” anywhere in the document. Of the 43 papers that conducted a cluster analysis, the most common clustering algorithm used was hierarchical clustering (60%) followed by k-means clustering (12%). In this review, the method that most resembled LDA was adopted to study population structure in microsatellite data , using a method originally developed by . This method is similar to LDA in the sense that individual genotype is represented as a mixture of the genotype of ancestral populations. Yet, to our knowledge, this method has been used predominantly for genetic data. Finally, several researchers have analyzed species compositional data for different purposes (e.g., to understand how organisms use resources and reconstruct historical forest composition based on pollen sediments), often relying on the Dirichlet-Multinomial or the logistic normal distribution . Yet, these studies fundamentally differ from ours in that their goal was not to identify biological communities in species-rich datasets.

In summary, we believe that the combination of several features (e.g., allowing for a sampling unit to be composed of multiple component communities, the ability to account for missing data and report uncertainty estimates) into a single tool is what makes LDA an innovative method relative to other existing clustering methods.

**2. Simulations to compare LDA to more commonly used clustering methods**

Clustering methods are typically used as a dimension reduction tool for community ecology, enabling researchers to interpret results based on a much smaller number of groups than the original number of species. To compare LDA to some of the existing clustering methods, we asked ourselves: how many clusters do these existing methods have to create to fit the data as well as LDA? A good method would ideally fit well the data with the smallest number of groups possible, thus aiding the interpretation of its outcomes.

To this end, we created four distinct datasets, assuming that 5%, 33%, 66%, and 95% of the sampling units were mixed units (i.e., were composed of multiple component communities). We assumed there were 1,000 sampling units, with 100 individuals each and a total of 200 species. Similar to the simulation reported in the main manuscript, we assumed that the true number of component communities was equal to 3. The relative abundance of each species in component community j, summarized in the vector
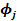
, was generated from a Dirichlet(1) distribution (i.e.,
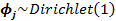
). Similarly, for the sampling units that were mixed, the relative abundance of each community in sampling unit l, summarized in the vector
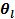
, was generated from the same distribution (i.e.,
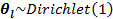
).

We fitted each dataset using three commonly used clustering methods: hierarchical (HC), k-means (KM), and model-based clustering (MC). We allowed the number of groups to vary from 3,…,10 for each of these clustering methods. We also fitted each dataset with LDA, a priori assuming 3 groups. For the stochastic methods (i.e., LDA and MC), we ran these algorithms 10 times to have an estimate of the uncertainty of the goodness-of-fit. For k-means clustering, 25 random starts were used. We assessed how well these methods fitted the data by calculating the log-likelihood as our measure of goodness-of-fit. Let the number of individuals for species k in sampling unit l be denoted by
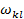
. We assume that the likelihood of the number of individuals by species in sampling unit l (i.e.,
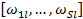
) is given by a multinomial distribution with size equal to
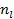
 (the number of individuals in this sampling unit) and probability vector given by
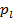
:


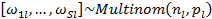


For the traditional clustering methods that we tested, we estimated
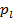
 in the following way. Assuming that sampling unit l was classified into cluster j, we set
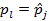
, where the element of this vector corresponding to species k (i.e.,
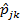
) is given by the proportion of individuals from species k in all sampling units classified into cluster j. For LDA, we estimated
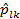
 as
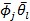
, where
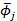
 and
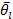
 are the mean of the posterior distribution of these parameters.

Using the LDA results with three groups as a benchmark, we assessed how many groups would be required for the other methods to fit the data as well as LDA. We find that many more groups are needed for these more traditional clustering methods to fit the data as well as LDA with only three groups. For instance, hierarchical clustering (HC) cannot fit the data as well as LDA even using 10 clusters for the data with 33% or more of mixed sampling units. K-means clustering (KM) performs slightly better than HC but exhibits similar problems (Fig. S1). Model-based clustering (MC) was consistently the worse clustering method and therefore is not shown in Fig. S1. We also compared the performance of these methods using other metrics, such as sum of squared errors
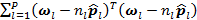
 and the Bray-Curtis distance
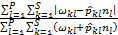
. LDA outperformed HC, KM and MC, for all goodness-of-fit metrics and all datasets (data not shown). There was only one exception to this pattern; the only time when LDA performed worse than HC and KM with three groups was using the Bray-Curtis distance as a measure of fit for the data with only 5% of mixed sampling units.

In hindsight, it is obvious that LDA would yield better results because it has many more parameters than the more traditional clustering methods. For instance, if there is a sampling unit that does not match any of the component communities, LDA can still try to fit it by combining these component communities in different proportions. On the other hand, traditional clustering methods are stuck having to assign this sampling unit to one of the existing clusters. Yet, despite the greater number of parameters, results from LDA with three groups are as easy to interpret as those from the other clustering methods with three groups.

Another alternative approach to compare these different clustering methods would have been to conduct a cross-validation exercise. This approach would avoid the problem of comparing methods with different underlying number of parameters by focusing on out-of-sample predictive skill. However, we did not pursue this approach because it is not straight-forward to make predictions for out-of-sample observations based on these traditional clustering methods.

**3. Implementing the Latent Dirichlet Allocation (LDA) model**

To describe how LDA is used to fit the data, we have to start by first describing its generative model. We then detail how some of the parameters can be integrated out, resulting in a collapsed model. Finally, we describe the full conditional distributions used in our Gibbs sampler, which are identical to those in . We end this section with a description of two important LDA operations. The first operation estimates the relative abundance of pre-determined component communities for new sampling units (i.e., the folding-in operation), which is particularly useful when assessing temporal changes. The second operation is focused on data imputation, which treats missing data as additional parameters to be estimated.

*3.1. Generative model*

We start by assuming that we have C communities, P sampling units, and S unique species. Let
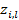
 be the latent variable indicating the community from which the i-th individual in the l-th sampling unit was drawn. Let
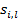
 be the species for this individual. Then,
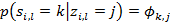
 is the probability of species k in community j. Furthermore,
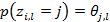
 denotes the probability of community j in sampling unit l.

The probability of each community in sampling unit *l* is given by the vector
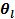
**,** drawn from:


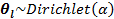


Similarly, the probability of each species in community *j* is given by the vector
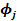
**,** drawn from:


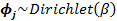


For each individual *i* in sampling unit *l*, we sample its community
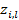
 and species
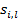
 from the following distributions:


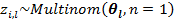


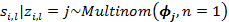


*3.2. Collapsed model*

To make the model fitting algorithm more efficient, we can integrate out the parameters
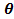
 and
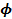
 in the following way:


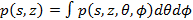


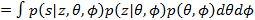


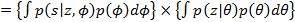
 [eqn. 1]

We start with the first term in eqn. 1 for all the individuals assigned to community j:


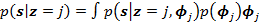


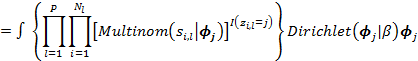


where
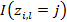
 is the indicator function, returning 1 if the condition is true and zero otherwise, and
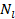
 is the number of individuals in sampling unit *l*. Because the multinomial and the Dirichlet distributions are conjugate, this integral is available in closed form, being equal to:


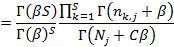


where
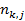
 is the number of individuals of species *k* in community *j* and
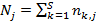
. We then extend this expression to all the communities, yielding:


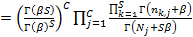
 [eqn. 2]

Now we tackle the last term in eqn. 1. Here, we focus on all individuals from sampling unit *l*:


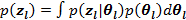


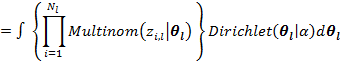


Again, because of conjugacy, this integral simplifies to:


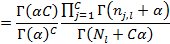


where
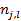
 is the number of individuals in community *j* in sampling unit *l* and
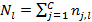
. Then, we extend this expression to all sampling units, yielding:


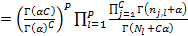
 [eqn. 3]

Thus, the joint distribution for the collapsed model described in eqn. 1 is given by combining the results in eqn. 2 and eqn. 3:


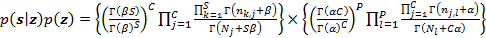
 [eqn. 4]

*3.3. Full conditional distributions*

To be able to use the Gibbs sampler for this problem, we have to find the full conditional distributions for each element of the vector
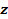
given all the other elements contained in this vector and the data
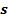
. This full conditional distribution is given by


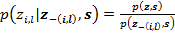
 [eqn. 5]

where
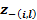
is the **z** vector without the element
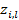
. Let the community, species, and sampling unit from the removed
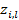
 be denoted by
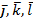
, respectively. The derivation of the denominator
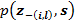
 is similar to that for
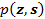
, thus we do not provide more detail here and just note that it can be shown to be equal to:


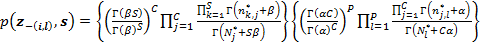
 [eqn. 6]

where
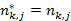
 for all k except for
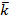
, in which case we have
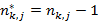
. Similarly,
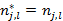
 and
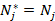
 for all j except for
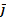
, in which case we have
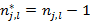
 and
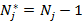
, respectively. Finally,
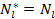
 for all l except for
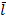
, in which case
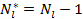
.

Combining the results in equations 4 and 6 to form the full conditional distribution in eqn. 5 yields:


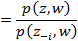


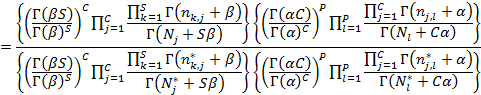


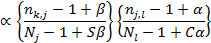


We can simplify this expression a bit more by noting that
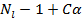
 does not depend on
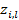
, yielding:


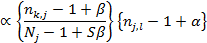


*3.4. Algorithmic details*

Our Gibbs sampler systematically cycles through all the elements
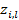
, updating these parameters one at a time. We sample each
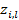
 from a multinomial distribution with n=1 and with probability vector
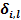
 given by:


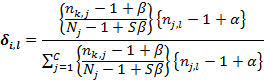


Despite the fact that we have integrated out the parameters
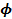
 and
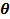
, we can still generate the posterior distribution for these parameters by deriving their full conditional distributions, given by:


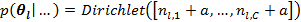


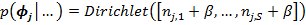


Given the large number of parameters in this model, it is not feasible to look at trace-plots from individual parameters. Rather, we assess convergence through the trace plot of the log-likelihood.

*3.5. Priors*

The parameters
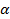
 and
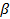
 are the hyper-prior parameters for LDA. In general, bigger
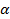
 and
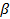
 tend to induce more smoothness (i.e., species tend to have more similar relative abundances in each component community and communities tend to have more similar relative abundances in each sampling unit). The parameter
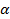
 and
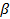
 are typically set to one in statistical models that adopt a Dirichlet prior distribution because this choice results in a uniform prior within the simplex (i.e., ), resulting in parameter estimates that are directly comparable to those from Maximum Likelihood Estimation. These are the hyper-parameter values that we adopt throughout this article, as in Griffiths and Steyvers (2004). However, we do acknowledge that this prior can be made less informative by choosing smaller positive values of
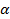
 and
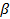
, as evident from the full conditional distributions of the parameters
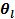
 and
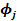
 given above. An alternative promising idea would have been to estimate
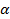
 and
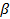
 as well by adding another layer to our hierarchical Bayesian model.

*3.6. Folding-in operation*

We can use the results from this model to estimate the relative abundance of pre-determined component communities. More specifically, we estimate community composition of new sampling units by estimating the matrix
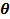
while keeping the matrix
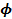
constant (see Figure 1 in the main text), in an operation known as folding-in. This is often done in text-mining to classify new documents in relation to topics that have already been created. To implement this prediction operation, we have to sample all
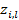
 and while conditioning on all (we are assuming these parameter remain fixed). Again, we rely on a Gibbs sampler for this.

The full conditional distributions for is given by a multinomial distribution with n=1 and probability vector . The probability for community j is given by:

The full conditional distribution for is the same as described previously.

*3.7. Data imputation operation*

We treat missing data as parameters to be estimated. As a consequence, our imputation method is completely coherent with our biodiversity analysis. Say data from sampling unit *l* are given by , where data for individuals are missing (NA stands for Not Available). We know that the reason for not measuring these individuals was that they were not in the species list of the study. More specifically, say that the species list contained species 1,…,k and the missing species were k+1,…,S. When we impute the species , we have to select a species from k+1,…,S. This is done by imputing from the conditional multinomial distribution for the missing species . In this distribution, the factor normalizes the probability vector to sum to one.

Thus, when data are missing, our Gibbs sampler has to be modified to also sample all missing species . We start by completing the dataset so that we have all . Then, our Gibbs sampler iterates by:

- Sampling all from
- Sampling for all component communities (j=1,…,C) from
- Sampling for all sampling units (l=1,…,P) from
- Sampling all missing data (i.e., for ) from

**4. Description of the analysis using standard multivariate methods**

Cluster analysis is the traditional multivariate method used in ecology to identify groups without *a priori* knowledge of how many groups exist. Ecologists employ many different types of cluster analyses and use several methods for testing the validity of the groups identified by cluster analysis, including the analysis of similarity and the multi-response permutation procedure . Here we take four traditional approaches to identifying and testing groups within our simulated dataset (details regarding how these dataset were created are given in the main text).

*4.1. Hierarchical clustering and MRPP*

Hierarchical clustering is a common clustering technique with the goal of hierarchically arranging entities into clusters based on dissimilarity. We transformed the raw simulation data matrix into a Bray-Curtis distance matrix and used the “average” linkage criterion in *hclust* (stats package R *v*.3.0.2). We then “cut” the dendrogram (function *cutree*, vegan package R *v*.3.0.2) to delineate either 3, 4, 5, and 6 groups (data not shown) and tested the significance of these groups using MRPP (vegan package) with 999 permutations. The MRPP resulted in significant differences (P < 0.01) for all groupings and the “chance-corrected agreement within groups”, *A*, was > 0.2 for all groupings. The three and four group dendrograms contain groups with over thirty sampling units. On the other hand, when we cut the dendrogram into five or six groups, the analysis identifies four groups that contain more than 30 sampling units while the remainder contains two or less. We do not view these groups with few sampling units as ecologically relevant. Taken together, we interpret the hierarchical cluster analysis and MRPP results as indicating that there are four groups.

*4.2. Hierarchical clustering and ANOSIM*

ANOSIM is another permutation-based significance test that uses ranked dissimilarities to test for the existence of groups and is often used in conjunction with Non-Metric Multidimensional Scaling (NMDS). ANOSIM requires an *a priori* selection of groups, so we used the groups identified by the cutting of the hierarchical cluster analysis to both run ANOSIM and plot the groups in NMDS ordination space. Groups of 3, 4, 5, and 6 (Fig. S2) were all found to be significantly different (P < 0.01) and the *R* statistic was > 0.95 in all cases indicating that pairs of samples within each group are more similar to each other than pairs of samples outside the group. The results are similar to that of the MRPP in terms of the number of sampling units in each group as we increased the number of groups. The three and four group NMDS plots contain groups with over thirty sampling units (Fig. S2). However, when we partition the plots into five or six groups, we find four groups that contain more than 30 sampling units while the remainder contains two or less. Overall, our interpretation is that this analysis suggests four groups. Similarly to the hierarchical clustering and MRPP approach, additional groups were found to be significant, but we do not view these as ecologically relevant given that they contained very few sampling units.

*4.3. Model-based clustering*

Model-based clustering provides a formal model describing the clustered structure of the data. By postulating a model, this class of clustering techniques allows for formal inference to be drawn with regards to the number of clusters that best describe a data set. We used a finite mixture model, which assumes that the data set contains groups that each have a different multivariate probability density function. Estimating the parameters of the mixture of probability density functions for the entire data set allows for the calculation of posterior probabilities of cluster membership . We used the function *Mclust* (mclust package R *v*.3.0.2) to cluster our simulation data and used the Bayesian Information Criterion (BIC) to determine the best fit model and thus the optimal number of clusters. The best fit model contained five clusters (Fig. S3). However the five cluster model included a group with one sampling unit, which we view as not ecologically relevant, and interpret the model-based clustering analysis as indicating four groups (Fig. S3).

*4.4. K-means clustering*

K-means clustering is a non-hierarchical divisive clustering approach that requires *a priori* specification of the number of clusters. A centroid is assigned to each cluster and an iterative procedure assigns samples to cluster by minimizing the within-cluster sum-of-squares. We used the function kmeans (stats package R v.3.0.2) to cluster our simulation data. We selected the optimal number of cluster based on a scree plot of the within-cluster sum of squares and a silhouette plot for two to 10 clusters. The optimal number of clusters for the simulated data was three.

**5. Description of the R files for the analysis of the simulated data**

Here we briefly describe the R files that we make available. These files reproduce part of the simulated data results described in the main text. The simulated data is given in “fake data agg.csv”, which contains a matrix of 1000 rows (i.e., 1000 sampling units) by 200 columns (i.e., 200 species), where each cell corresponds to the abundance of individuals from that particular species in that particular sampling unit. The file “topicmodel simul data.r” contains R code that fits the LDA model to this simulated data using the package ‘topicmodel’. Based on the outcomes of the function ‘LDA’, it produces figures that resemble those in Figures 3C and 3D in the main manuscript.

**Supplementary Table 1: Most abundant characteristic species for each component community identified for eastern United States.**

| Community | Dominant characteristic species |
| --- | --- |
| 1 | *Acer rubrum* |
| 2 | *Pinus strobus, Tsuga canadensis, Betula lenta*, and *Quercus rubra* |
| 3 | *Pinus taeda* |
| 4 | *Quercus alba, Quercus prinus*, and *Quercus velutina* |
| 5 | *Fraxinus pennsylvanica* and *Ulmus Americana* |
| 6 | *Picea rubens, Betula alleghaniensis,* and *Fagus grandifolia* |
| 7 | *Picea mariana, Pinus echinata, Liquidambar styraciflua*, and *Quercus nigra* |
| 8 | *Juniperus virginiana, Acer saccharinum, Fraxinus americana*, and *Tilia americana* |
| 9 | *Liriodendron tulipifera* and *Prunus serotina* |
| 10 | *Abies balsamea, Pinus resinosa, Betula papyrifera*, and *Populus tremuloides* |
| 11 | *Pinus elliottii, Thuja occidentalis*, and *Nyssa biflora* |

**Supplementary Table 2: Characteristics of the Costa Rica dataset.** Characterization of each plot and size class in relation to time since abandonment, year of measurement, and monitored species.

| Sites | Years since abandonment | Year of measurement | Size class | Species |
| --- | --- | --- | --- | --- |
| BEJ | 10 | 2005 | small and large tree | All |
| CR | 25 | 1997 | small and large tree | All |
| JE | 10 | 2005 | small and large tree | All |
| LEP | 20 | 1997 | small and large tree | All |
| LEPviejo | Old Growth | 2005 | small and large tree | All |
| LSUR | 12 | 1997 | small and large tree | All |
| SV | Old Growth | 2005 | small and large tree | All |
| TIR | 15 | 1997 | small and large tree | All |
| BEJ | 11 | 2006 | Saplings | Just canopy tree species |
| JE | 11 | 2006 | Saplings | Just canopy tree species |
| LEP | 29 | 2006 | Saplings | Just canopy tree species |
| LEPviejo | Old Growth | 2006 | Saplings | Just canopy tree species |
| LSUR | 21 | 2006 | Saplings | Just canopy tree species |
| SV | Old Growth | 2006 | Saplings | Just canopy tree species |

**Supplementary Table 3: Most abundant characteristic species for each component community identified for the Costa Rica dataset.**

| - Community | Dominant characteristic species |
| --- | --- |
| 1 | *Protium pittieri*, *Protium ravenii*, *Faramea parvibractea*, *Euterpre precatoria* var. *longevaginata*, *Iriartea deltoidea*, and *Welfia regia* |
| 2 | *Cordia alliodora, Inga cocleensis, Miconia elata, Simarouba amara*, *Byrsonima arthropoda*, and *Vochysia ferruginea* |
| 3 | *Xylopia sericophylla, Miconia affinis, Cordia bicolor, Casearia arborea, Laetia procera*, and *Goethalsia meiantha* |

**Supplementary Figure Legends**

**Figure S1**. LDA with 3 component communities performs substantially better than more commonly used clustering methods. The title of each panel refers to the percentage of sampling units that are mixed (i.e., have individuals from more than one component community). K-means clustering (black lines and circles) typically performs better than hierarchical clustering (red lines and circles), yet requires many more clusters to fit the data as well as LDA with 3 component communities (blue circle), particularly as the proportion of mixed sampling units increases. Results for model-based clustering are not shown because it had a substantially worse performance when compared to all the other methods. LDA results are based on the mean of 10 runs because the results from the individual runs were very similar and would have been indistinguishable in this plot.

**Figure S2:** NMDS plots constructed using Bray-Curtis distance. The groups from the hierarchical cluster analysis were used to run an ANOSIM and color convex hulls in the NMDS plots. The three, four, five, and six groups were found to be significantly different. Similar to the MRPP approach, the five and six group plots only have four clusters with more than two sampling units.

**Figure S3:** Clusters from the best-fit finite mixture model identified using the Bayesian Information Criterion (BIC). The clusters are plotted against the first two principal components of the simulated data set. Although the five cluster solution was identified as the best fit model, one of the five clusters contains only one data point (red point).

**Fig. S1**

**Fig. S2**

**Fig. S3**

References

1.

Agarwal, S., Lim, J., Zelnik-Manor, L., Perona, P., Kriegman, D. & Belongie, S. (2005). Beyond pairwise clustering. *Proc CVPR IEEE*, 2, 838-845.

2.

Billheimer, D., Guttorp, P. & Fagan, W.F. (2001). Statistical interpretation of species composition. *J Am Stat Assoc*, 96, 1205-1214.

3.

Clarke, K.R. (1993). Non-parametric multivariate analysis of changes in community structure. *Aust J Ecol*, 18, 117-143.

4.

de Valpine, P. & Harmon-Threatt, A.N. (2013). General models for resource us or other compositional count data using the Dirichlet-multinomial distribution. *Ecology*, 94, 2678-2687.

5.

Everitt, B. & Hothorn, T. (2011). *An introduction to applied multivariate analysis with R*. Springer, New York, NY.

6.

Fortunato, S. (2010). Community detection in graphs. *Phys Rep*, 486, 75-174.

7.

Fraley, C. & Raftery, A.E. (1998). How many clusters? Which clustering method? Answers via model-based cluster analysis. *Comput J*, 41, 578-588.

8.

Fraley, C. & Raftery, A.E. (2002). Model-based clustering, discriminant analysis, and density estimation. *J Am Stat Assoc*, 97, 611-631.

9.

Griffiths, T. & Steyvers, M. (2004). Finding scientific topics. *Proc Natl Acad Sci U S A*, 101, 5228-5235.

10.

Jain, A.K. (2009). Data clustering: 50 years beyond K-means. *Pattern Recogn Lett*, 31, 651-666.

11.

McCune, B., Grace, J.B. & Urban, D.L. (2002). *Analysis of ecological communities*. MjM Software Design, Gleneden Beach, Oregon.

12.

Paciorek, C.J. & McLachlan, J.S. (2009). Mapping ancient forests: Bayesian inference for spatio-temporal trends in forest composition using the fossil pollen proxy record. *J Am Stat Assoc*, 104, 608-622.

13.

Pritchard, J.K., Stephens, M. & Donnelly, P. (2000). Inference of population structure using multilocus genotype data. *Genetics*, 155, 945-959.

14.

Razgour, O., Juste, J., Ibanez, C., Kiefer, A., Rebelo, H., Puechmaille, S.J. *et al.* (2013). The shaping of genetic vatiation in edge-of-range populations under past and future climate change. *Ecol Lett*, 16.
